# Cholinergic Stimulation Exerts Cardioprotective Effects and Alleviates Renal Inflammatory Responses after Acute Myocardial Infarction in Spontaneous Hypertensive Rats (SHRs)

**DOI:** 10.3390/ph17050547

**Published:** 2024-04-24

**Authors:** Pamela Nithzi Bricher Choque, Maria Helena Porter, Manuella S. Teixeira, Humberto Dellê, Rosilene Motta Elias, Bruno Durante, Marina Rascio Henriques Dutra, Christine N. Metz, Valentin A. Pavlov, Fernanda M. Consolim Colombo

**Affiliations:** 1 Department of Medicine, Universidade Nove de Julho (Uninove), São Paulo 01504-001, SP, Brazil; pamelabricher@gmail.com (P.N.B.C.); maria.fonseca@online.uscs.edu.br (M.H.P.); hdelle@uni9.pro.br (H.D.); rosilene.elias@uni9.pro.br (R.M.E.); dutra.marinahenriques@gmail.com (M.R.H.D.); 2 Hypertension Unit, Heart Institute, Medical School, University of São Paulo, São Paulo 05403-900, SP, Brazil; manuella.teixeira@usp.br (M.S.T.); durante.bruno@gmail.com (B.D.); 3 The Feinstein Institutes for Medical Research, Northwell Health, Manhasset, NY 11030, USA; cmetz@northewell.edu (C.N.M.); vpavlov@northwell.edu (V.A.P.); 4 Donald and Barbara Zucker School of Medicine at Hofstra/Northwell, Hempstead, NY 11550, USA

**Keywords:** acute myocardial infarction, cardiorenal syndrome, chronic kidney injury, inflammation, autonomic nervous system, cholinergic stimulation, pyridostigmine bromide, vagus nerve

## Abstract

Background: In this investigation, we explored the effects of pharmacological cholinergic stimulation on cardiac function and renal inflammation following acute myocardial infarction (AMI) in spontaneously hypertensive rats (SHRs). Methods: Adult male SHRs were randomized into three experimental groups: sham-operated; AMI + Veh (infarcted, treated with vehicle); and AMI + PY (infarcted, treated with the cholinesterase inhibitor, pyridostigmine bromide (PY)—40 mg/kg, once daily for seven days). Rats were euthanized 7 or 30 days post-surgery. The clinical parameters were assessed on the day before euthanasia. Subsequent to euthanasia, blood samples were collected and renal tissues were harvested for histological and gene expression analyses aimed to evaluate inflammation and injury. Results: Seven days post-surgery, the AMI + PY group demonstrated improvements in left ventricular diastolic function and autonomic regulation, and a reduction in renal macrophage infiltration compared to the AMI + Veh group. Furthermore, there was a notable downregulation in pro-inflammatory gene expression and an upregulation in anti-inflammatory gene expression. Analysis 30 days post-surgery showed that PY treatment had a sustained positive effect on renal gene expression, correlated with a decrease in biomarkers, indicative of subclinical kidney injury. Conclusions: Short-term cholinergic stimulation with PY provides both cardiac and renal protection by mitigating the inflammatory response after AMI.

## 1. Introduction

Acute myocardial infarction (AMI) remains a leading cause of morbidity and mortality on a global scale [1]. AMI produces localized cardiac injury and initiates a systemic inflammatory cascade that may lead to multi-organ dysfunction [2]. Renal involvement following AMI is particularly concerning as it substantially worsens the prognosis [3]. The interplay between the primary cardiac injury in AMI and acute kidney injury is a critical determinant of patient outcomes [4], highlighting the urgent need for novel therapeutic strategies to mitigate this inter-organ crosstalk [5,6,7].

The pathophysiological mechanisms which link AMI to acute kidney injury are multifactorial and complex, encompassing hemodynamic alterations, neurohormonal activation, and inflammatory pathways [7]. Central to the inflammatory response is the uncontrolled production of pro-inflammatory cytokines, including tumor necrosis factor (TNF), interleukins (IL-1β, IL-6), and a cascade of other inflammatory mediators, which together orchestrate the acute phase response and contribute to renal parenchymal injury [8,9]. The ensuing inflammatory milieu not only exacerbates cardiac damage post-AMI, but also drives a deleterious cycle of renal endothelial injury, tubular cell apoptosis, and fibrosis, leading to chronic kidney injury [6].

Amidst the search for new interventions for treating excessive and chronic inflammation, there is a growing interest in the neural regulation of inflammatory responses and, specifically, in the cholinergic anti-inflammatory pathway [10]. This vagus nerve-based pathway controls inflammation through cholinergic signaling, activating the alpha7 nicotinic acetylcholine receptor (α7nAChR) expressed on macrophages and other immune cells [11]. Electrical vagus nerve stimulation and cholinergic modalities, including α7 nAChR agonists and cholinesterase inhibitors have been successfully explored for treating a variety of inflammatory conditions [12,13,14], including kidney diseases [15,16,17,18,19].

Pyridostigmine (PY), a reversible inhibitor of cholinesterase enzymes which catalyze acetylcholine degradation, is a cholinergic drug which can enhance cholinergic signaling, including efferent vagus nerve activity [20]. The anti-inflammatory efficacy of PY after AMI has been previously reported [20,21,22]. However, its renal-specific anti-inflammatory effects in the setting of AMI-induced AKI remain poorly investigated. This gap in knowledge is particularly pertinent given the increasing prevalence of AMI-induced AKI and the paucity of effective treatments for this condition.

In this study, we focused on elucidating the effects of PY on cardiac function and renal inflammation and injury in the context of AMI, utilizing a rat model which closely resembles human pathophysiology–spontaneous hypertensive rats (SHRs). We found that short-term (7 day) treatment with PY, following AMI in SHRs, had immediate cardioprotective and renal anti-inflammatory effects. Importantly, a long-term follow-up at 30 days, revealed the sustained beneficial impact of PY treatment. These findings are of significant interest for future preclinical and clinical studies exploiting PY cholinergic stimulation in the context of AMI and renal injury.

## 2. Results

### 2.1. Alterations in Hemodynamic Indices and Cardiovascular Variability during AMI and Effects of Pyridostigmine

As illustrated in Table 1, the hemodynamic parameters across the three groups were evaluated at two time points—7 days and 30 days post-AMI or sham surgery. After 7 days, there was a notable reduction in systolic blood pressure (SBP), diastolic blood pressure (DBP), and mean arterial pressure (MAP) in the AMI + Veh group compared to the sham group, indicating the early hemodynamic impact of AMI. Notably, the AMI + PY group exhibited a more pronounced decrease in both DBP and MAP than the AMI group, suggesting an additive effect of PY on these parameters. However, the reduction in SBP was not statistically significant in the AMI + PY group when compared to the AMI + Veh group. Heart rate (HR) was increased in the AMI + Veh group compared to the sham group, but this increase was not statistically significant when compared to the AMI + PY group, which maintained a HR comparable to the sham.

After 30 days, the sham group exhibited a slight increase in SBP and DBP, which remained within the normal physiological ranges. The SBP and MAP of the AMI + Veh group did not differ significantly from the sham group, indicating a possible recovery or adaptation over time. Conversely, the AMI + PY group presented significantly lower SBP and MAP values compared to the sham and AMI + Veh groups, reinforcing that PY treatment had a sustained impact on blood pressure regulation post-AMI. The HR among all groups at the 30-day evaluation did not show significant differences, likely indicating the stabilization of the cardiac rhythm following the acute phase of AMI.

As shown in Table 2, the components of heart rate and blood pressure variability, along with baroreflex sensitivity, were also evaluated in the three groups 7 days and 30 days post-AMI or sham surgery. After 7 days, a significant divergence in autonomic regulation was observed in the AMI + Veh group compared to the sham group. This was evidenced by an elevation in the low-frequency normalized units (LF nu) and a reduction in the high-frequency normalized units (HF nu), leading to an increased LF/HF ratio indicative of heightened sympathetic dominance. In contrast, the AMI + PY group showed a significant increase in parasympathetic activity, as revealed by the elevated values of the root mean square of successive differences between normal heart beats (RMSSD), absolute power of the heart rate variability (VARPI), and HF absolute (HF ab), and a return towards baseline values in the LF nu, HF nu, and LF/HF ratio. These findings suggest a modulation of the cardiac autonomic tone towards parasympathetic predominance in the PY-treated group, which is further corroborated by the augmented alpha index, a marker of baroreflex sensitivity. In terms of systolic blood pressure variability (SBPV), both AMI groups showed a reduced variability compared to the sham group, as indicated by the lower absolute power of blood pressure variability (VAR-SBP) and the absolute power of the low-frequency band of systolic blood pressure variability (LF-SBP) values. This could reflect an impaired autonomic response to blood pressure regulation post-infarction.

When evaluated after 30 days post-surgery, the sham and AMI groups presented comparable metrics across all parameters of HRV and BPV. This suggests an adaptive normalization over the month post-infarction. However, the AMI + PY group showed an enhanced profile with an increased total HR variability (as indicated by a higher VARPI) and an improved alpha index when compared to the sham group. These findings suggest a sustained, albeit modest, benefit from the PY treatment on autonomic function and cardiovascular regulation. Together, these findings underscore the potential of PY cholinergic treatment in modulating autonomic dysfunction post-AMI, with implications for improving cardiovascular outcomes in this model.

### 2.2. Alterations in Structural and Functional Echocardiographic Parameters during AMI and Effects of Pyridostigmine

As shown in Table 3, at 7 days post-AMI, rats with AMI treated with vehicle exhibited significant alterations in cardiac structure and function compared to the sham group. These included increased left ventricular systolic (LVSD) and diastolic diameters (LVDD), accompanied by a marked reduction in left ventricular ejection fraction (LVEF) and fractional area change (LVFAC), compared to the SHAM group. The elevated E/A ratio in the AMI group points towards diastolic dysfunction. In comparison, the AMI + PY group demonstrated some improvement, while the systolic function parameters (LVEF and LVFAC) did not significantly differ from the AMI + Veh group. There was evidence of improved diastolic function, indicated by a lower E/A ratio, as well as higher E′/A′ and lower E/E′ ratios when compared to the sham group, suggesting enhanced left ventricular relaxation.

At the 30-day assessment, the AMI + Veh group continued to show cardiac remodeling when compared to the sham group, similar to the 7-day findings, with further increased left ventricular diameters. In this later stage, the AMI + PY group did not exhibit a significant difference in systolic function parameters when compared to the AMI + Veh group. Also, the diastolic function parameters were not improved, contrary to the 7-day findings, with the E/A ratio, E′/A′ ratio, and E/E′ ratio resembling those of the AMI Veh-treated group, indicating that the early diastolic improvements observed in the AMI + PY group were not sustained over the 30-day period. Collectively, these observations suggest a potential role for PY treatment in ameliorating early diastolic dysfunction post-AMI and the lack of sustained diastolic improvement at 30 days.

### 2.3. Alterations in Renal Function Markers during AMI and Effects of Pyridostigmine

**Figure 1:**
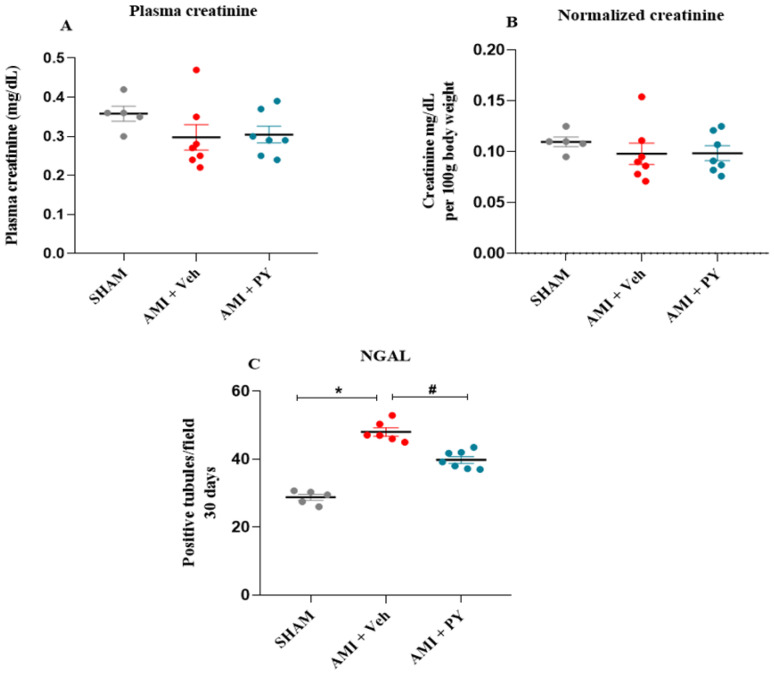
Plasma creatinine in the studied groups and quantification of the NGAL marker in the renal cortex. (**A**) Plasma creatinine measured 30 days post-sham surgery or AMI (±PY). (**B**) Normalized creatinine per 100 g of body weight measured 30 days post-sham survery or AMI (±PY). Values are presented as the mean ± standard error of the mean. (**C**) Bar graphs of NGAL positive tubules, measured 30 days post-sham or AMI (±PY). Values are presented as the mean ± standard error of the mean (SEM). * *p* < 0.05 when compared to the SHAM group. # *p* < 0.05 when compared to the AMI + Veh group. (*n* = 5–7, for each group).

Plasma creatinine and Neutrophil gelatinase-associated lipocalin (NGAL) expression in the renal cortex in the three groups of animals after 30 days post-surgery are shown in Figure 1. There were no significant differences in both plasma creatinine concentrations (Figure 1A) and plasma creatinine levels normalized to the body weight (Figure 1B) among all groups evaluated at this moment. NGAL (Figure 1C) is a marker of subclinical kidney injury [23] and its expression can indicate early damage to the renal tubules. Compared to the control group, AMI + Veh-treated rats had significantly higher numbers of tubules positively stained for NGAL. Pyridostigmine treatment significantly decreased these values.

**Figure 2:**
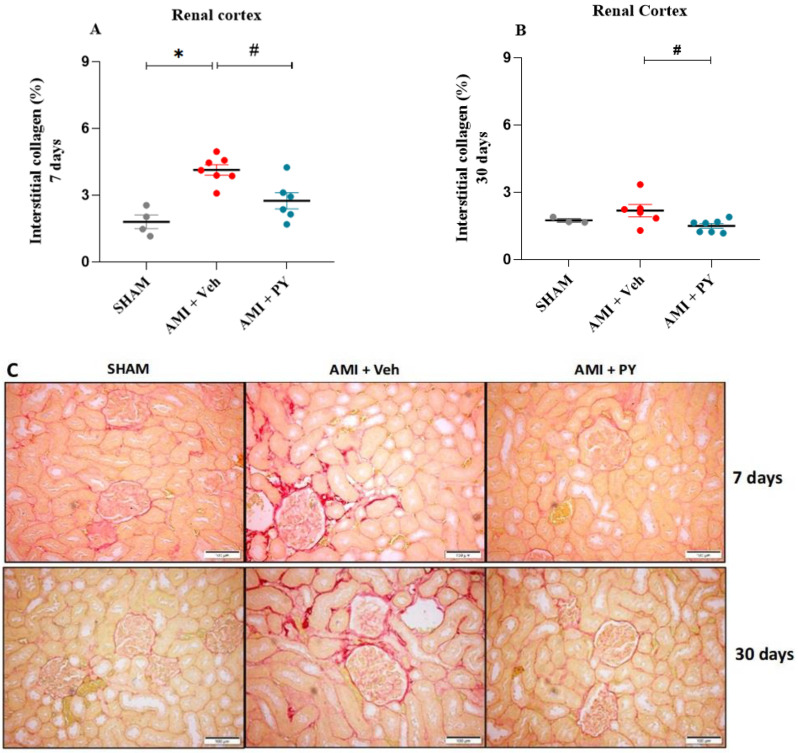
Quantification of interstitial fibrosis in the renal cortex by Picrossirius Red staining 7 and 30 days post-sham or AMI surgery. (**A**) percentage of interstitial collagen 7 days in the Sham, AMI + Veh and AMI + PY groups. Values presented as the mean ± standard error of the mean. * *p* < 0.05 when compared to the SHAM group. # *p* < 0.05 when compared to the AMI + Veh group. (**B**) percentage of interstitial collagen 30 days in the Sham, AMI + Veh and AMI + PY groups. Values presented as the mean ± standard error of the mean. * *p* < 0.05 when compared to the SHAM group. # *p* < 0.05 when compared to the AMI + Veh group. (**C**) Representative photomicrographs indicate the positive staining of collagen fibers in the kidney stained with Picrosirius red 7 and 30 days of protocol (scale bars = 100 µm). (*n* = 4–7, for each group).

As depicted in Figure 2, the AMI + Veh group showed significantly higher collagen levels in the kidneys compared to the sham group, when measured 7 days post-surgery. Moreover, PY-treated AMI rats exhibited significantly less collagen compared to AMI-Veh-treated animals, with collagen levels comparable to sham animals. Evaluating rats after 30 days revealed lower amounts of collagen in both AMI groups compared to the 7-day time point, with AMI-PY rats showing significantly lower levels compared to AMI-Veh rats. These observations indicate a positive residual effect of the PY treatment on kidney damage and collagen accumulation. 

### 2.4. Changes in CD68+ Macrophages in Renal Tissue following AMI and Effects of Pyridostigmine

**Figure 3:**
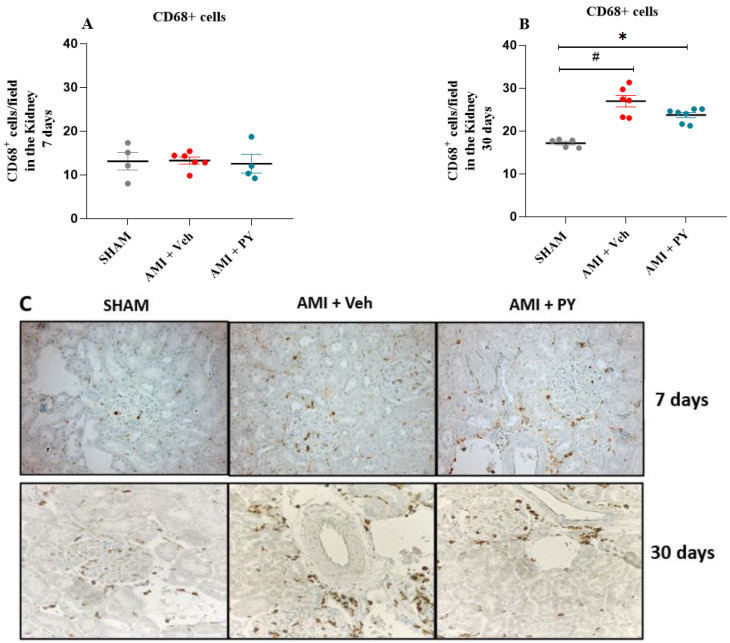
Quantification of CD68+ Cells (Total Macrophages) 7 and 30 days post-sham or AMI surgery. (**A**). Bar graphs of CD68+ positive macrophages 7 days in each group. Values presented as mean ± standard error of the mean. * *p* < 0.05 when compared to the Sham group. # *p* < 0.05 when compared to the AMI + Veh group. (*n* = 4–7, for each group). (**B**). Bar graphs of CD68+ positive macrophages 30 days in each group. Values presented as mean ± standard error of the mean. * *p* < 0.05 when compared to the Sham group. # *p* < 0.05 when compared to the AMI + Veh group. (*n* = 4–7, for each group). (**C**) Photomicrographs showing the total macrophages in the kidney tissue (per high power field), when assessed 7 and 30 days post-sham or AMI surgery (magnification 400×).

Next, we analyzed the effects of PY on macrophage infiltration into renal tissue during AMI. Representative photomicrographs (Figure 3C) illustrate the presence of CD68+ macrophages within renal tissues of the three groups of rats at 7 days and 30 days post-surgery. At 7 days post-surgery, the quantification of CD68+ macrophages within renal parenchyma did not reveal significant differences among the studied cohorts (Figure 3A). However, after 30 days post-AMI-surgery, renal specimens procured from the AMI rats showed a pronounced increase in CD68+ macrophage numbers when compared to the sham group and the administration of PY post-AMI decreased CD68+ macrophage numbers (Figure 3C). These observations indicate the inhibitory effect of PY on macrophage infiltration into the kidney. 

### 2.5. Changes in the Gene Expression of Pro-Inflammatory and Anti-Inflammatory Markers in Renal Tissue Post-Acute Myocardial Infarction and Effects of Pyridostigmine

**Figure 4:**
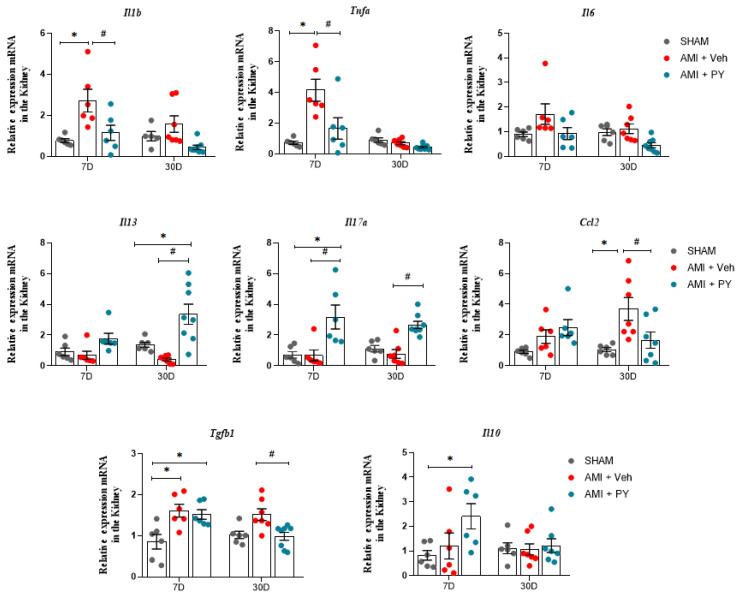
Analysis of mRNA expression of pro-inflammatory and anti-inflammatory genes in renal tissue at 7 days (7 D) or 30 days (30 D) after sham or AMI surgery. Values expressed as the mean ± SEM. ANOVA two way, followed by Tukey’s post-hoc test. * *p* < 0.05 when compared to the SHAM group; # *p* < 0.05 when compared to the AMI group, (*n* = 6–8, for each group).

We also analyzed the mRNA expression levels of pro-inflammatory and anti-inflammatory genes, including Il1b (encodes IL-1β), Tnfa (TNF-α), Il6 (IL-6), Ccl2 (MCP-1), Il13 (IL-13), Il10 (IL-10), and Tgfb1 or 2 or 3 (TGF-β) in renal tissues following acute myocardial infarction (AMI) in the three groups of rats. As shown in Figure 4, there was an increase in the renal mRNA expression of Il1b, Tnfa, and Tgfb in the AMI + Veh group relative to the sham group 7 days post-AMI. The mRNA expression levels of other inflammatory mediators did not exhibit notable differences. Animals with AMI and treated with PY showed significantly reduced renal mRNA expression levels of Il1b and Tnfa when compared to the AMI-Veh group and significantly increased renal mRNA expression of Il10 when compared to both the sham and AMI + Veh groups.

Further evaluation after 30 days-post-surgery demonstrated that renal mRNA expression levels of Il1b, Tnfa, and Il10 showed no significant differences across the three groups. However, the mRNA expression of Ccl2, which showed no differences at the 7-day assessment in the AMI group, was significantly elevated, compared to the sham group at 30 days. Similarly, Tgfb gene expression was also significantly higher in the AMI rats when assessed 30 days-post-surgery. Treatment with PY resulted in a persistent effect on gene expression profiles; AMI + PY-treated animals exhibited significantly reduced levels of Ccl2 and Tgfb mRNA expression in the kidneys, accompanied by increased Il13 mRNA expression in the kidneys, indicating the residual anti-inflammatory and immunomodulatory effects of PY.

## 3. Discussion

In this study, we demonstrated the therapeutic potential of PY in spontaneously hypertensive rats (SHRs) following acute myocardial infarction (AMI). Our findings suggest that a short-term treatment regimen with PY, lasting 7 days post-AMI, exerts acute cardioprotective effects and has a beneficial impact on mitigating AMI-induced acute renal inflammation. Furthermore, our results reveal a modest, yet statistically significant, sustained effect of PY on renal inflammation, indicating that PY holds considerable therapeutic promise for the management of post-AMI complications.

The interaction between the nervous and immune systems, in which vagus nerve cholinergic signaling plays a critical role, has garnered attention for the development of novel anti-inflammatory therapies [10,18]. The activation of this pathway suppresses the release of pro-inflammatory cytokines and inflammation, a process mediated through the α7nAChR expressed on macrophages and other immune cells [11,13]. Electrical vagus nerve stimulation or pharmacological cholinergic modalities, including α7nAChR agonists and cholinesterase inhibitors, have been successfully explored for the treatment of sepsis, arthritis, and other inflammatory conditions [14,24]. These preclinical studies have progressed to successful clinical trials in patients with metabolic syndrome, rheumatoid arthritis, inflammatory bowel disease, and other inflammatory conditions [25,26].

In our study we utilized the SHR model, which was developed in 1963 by Okamoto and Aoki, and has been extensively used to study arterial hypertension’s natural history, genetic factors, pathophysiological changes, and to evaluate new treatments [27,28]. SHRs exhibit an autonomic imbalance with increased sympathetic and reduced parasympathetic (vagal) activity, alongside chronic inflammation, closely resembling the clinical scenario in AMI patients.

A previous study demonstrated that the administration of PY for a period of 2 weeks in SHRs led to an increase in cardiac vagal modulation and a reduction in blood pressure, heart rate, and LVEF, as measured by radioisotopic ventriculography [29]. However, a more recent study investigated the effects of a lower dose of PY administered over the same 2-week treatment period. Interestingly, they observed similar hemodynamic and autonomic effects, without a decrease in cardiac function, as assessed by echocardiography [30]. The discrepancy in the effects of PY on cardiac function between these two studies may be attributed to the difference in the doses used and the methods employed to evaluate cardiac performance.

In SHRs, the post-MI heart might accelerate existing renal inflammation and fibrosis through systemic cytokine release. The efficacy of cholinergic stimulation in reducing hypertension-induced organ damage in normotensive and SHR models and in abdominal aorta coarctation-induced hypertension was demonstrated [31]. These models revealed deficiencies in cardiovascular tissue’s vesicular acetylcholine transporter and α7-nAChR, with an increase in pro-inflammatory cytokines. Chronic treatment with the α7nAChR agonist PNU-282987 improved endothelial function and reduced pro-inflammatory cytokines, indicating the role of cholinergic anti-inflammatory signaling in controlling endothelial dysfunction in hypertension [31]. Our findings show the beneficial efficacy of cholinergic stimulation in conditions of increased cardiovascular stress caused by AMI in SHRs.

AMI triggers systemic inflammation, potentially leading to multi-organ dysfunction [2]. Our results demonstrate that PY treatment stabilizes the heart rate and the sympathetic-vagal balance, indicating improved autonomic regulation in AMI rats. Additionally, we observed enhanced left ventricular diastolic function, highlighting cardioprotective effects of PY. Preclinical studies have shown that PY improves cardiac function and has anti-inflammatory effects immediately post-AMI in normotensive and SHR models [20,21,22]. However, no sustained cardioprotective effect was observed 30 days post-treatment. Our results indicate that a 7-day treatment with the cholinergic drug PY results in a significant shift towards parasympathetic (vagal) predominance in cardiac autonomic tone alongside an elevation in the alpha index, a marker of baroreflex sensitivity. The baroreflex is a crucial self-regulatory physiological cardiovascular mechanism [32], and a reduction in baroreflex sensitivity is closely correlated with increased mortality rates post-AMI, even among patients undergoing beta-blocker therapy [33]. Despite the recognized pathological significance of impaired baroreflex function in elevating post-AMI mortality rates, the search for effective long-term pharmacological interventions continues. Our observations suggests that PY treatment may improve cardiovascular autonomic balance and baroreflex sensitivity in AMI. In terms of systolic blood pressure variability (SBPV), a reduction in variability was noted in both AMI cohorts, as evidenced by lower VAR-SAP and LF-SAP values.

The anti-inflammatory effects of cholinergic stimulation, utilizing α7nAChR agonists such as nicotine and GTS-21 on AKI, have been demonstrated in several preclinical studies, primarily in models of sepsis-induced AKI and renal ischemia-reperfusion injury [19]. Electrical vagus nerve stimulation also results in a substantial reduction in both renal injury and systemic inflammation in renal ischemia-reperfusion injury in models [13,18,34]. These effects were not seen in α7nAChR knockout mice or splenectomized mice, indicating that the essential role of the α7nAChR and the spleen in the observed protective effects. The efferent vagus nerve does not innervate the kidney directly [35], suggesting that vagus nerve stimulation (VNS) appears to exert its reno-protective effect indirectly through the spleen. Our results importantly add to these observations by revealing the potential of cholinergic modulation, particularly through agents like PY, in mitigating renal inflammation and injury post-AMI.

Among the pathophysiological mechanisms that link AMI to AKI, aberrant renal inflammatory responses play an important role [7]. Renal macrophage infiltration is a key determinant of inflammatory responses. Upon initial assessment at the 7-day mark after AMI, the number of CD68+ macrophages within renal parenchyma did not present a significant difference among the studied groups. However, at 30 days post-AMI, renal specimens from the AMI rats manifested a pronounced augmentation in CD68+ macrophages compared to the sham group. Of note, the administration of PY post-AMI was correlated with a significant decrease in CD68+ macrophage numbers within renal tissue, underscoring the anti-inflammatory and tissue-protective effects of this drug [36].

The effects of PY on the renal gene expression profiles of key inflammatory molecules during AMI further substantiated its beneficial immunomodulatory and anti-inflammatory action. At 7 days, there was a substantial increase in the mRNA expression of Il1b (encodes interleukin-1 beta or IL-1β) and Tnfa (encodes tumor necrosis factor-alpha), compared to the sham group. This increase in renal cytokines following AMI corroborates previous studies in normotensive rats [8,9]. Importantly, animals with AMI and treated with PY exhibited significantly reduced mRNA expression levels of Il1b and Tnfa, alongside a heightened expression of interleukin-10 (Il10). At 30 days, mRNA expression levels of Il1b, Tnfa, and Il10 normalized and showed no significant differences across all groups. Treatment with PY had a persistent influence on gene expression profiles, beyond these cytokines. This suggests that PY not only tempers the acute inflammatory response post-AMI but also modulates the longer-term immune landscape within the kidney. The reduction in pro-inflammatory cytokines Il1b and Tnfa by PY, together with the increase in the anti-inflammatory cytokine Il10 in the AMI + PY group, highlight a shift in the inflammatory balance towards resolution and healing [37].

The expression of the Tgfb gene was observed to be elevated in AMI rats compared to the controls at the 7-day protocol, and PY treatment did not modify its expression at this time point. However, the expression of the Tgfb gene, while still notably higher in AMI rats at the 30-day protocol, was significantly decreased by PY treatment. TGF-β1, a pleiotropic cytokine, has been recognized as a pivotal mediator of kidney fibrosis. Recent evidence elucidates a complex framework of signaling networks that facilitate the multifunctionality of TGF-β1 actions, including the upregulation of NF-κB [37]. NF-κB, a transcriptional regulator, profoundly influences the inflammatory response and fibrosis. Dysregulation of the TGF-β/smad signaling pathway is identified as a potential pathogenic mechanism in hypertension-related renal damage [31]. Thus, the specific targeting of the TGF-β/NF-κB signaling pathway appears to be crucial and presents an appealing molecular therapeutic strategy. The Smad-dependent TGF-β signaling pathway is a major contributor to fibrogenesis in both the heart and kidney in the post-MI context [8]. The activation of this pathway in the kidneys of normotensive rats has been previously documented, starting a week post-MI [8]. PY physiological action is related to increasing the levels of acetylcholine. Acetylcholine has been reported to interact with the α7-nAChR on cytokine-producing immune cells, inhibiting the activation of NF-κB and the subsequent induction of a pro-inflammatory cascade [11]. A recent study demonstrated that the stimulation of the cholinergic anti-inflammatory pathway through the administration of GTS-21 protected against AngII-induced hypertension by enhancing autonomic control, suppressing NF-κB activation, and reducing renal fibrosis and the inflammatory response [38]. Our results indicate the potential of PY in reducing this signaling pathway after AMI, possibly through increasing acetylcholine levels.

The mRNA expression of Ccl2, which remained unchanged at the 7-day assessment in the AMI group, was found to be significantly elevated (compared to the sham group) at 30 days. Notably, PY-treated animals exhibited a significant reduction in Ccl2 gene expression. The monocyte chemoattractant protein-1 (MCP-1), also known as chemokine ligand 2 (CCL2), is a member of the extensive chemokine family and acts as a key mediator of innate immunity and tissue inflammation. CCL2 (MCP-1) directs neutrophils, monocytes, and T cells to sites of inflammation through chemokine receptor engagement and is secreted by various cells, including immune cells, endothelial cells, and renal tubular cells [39]. MCP-1 not only serves as an indicator of the occurrence, progression, and prognosis of disease but is also intricately linked with the severity and stage of nephropathy. Upon stimulation, or severe damage to renal tissue, MCP-1 expression increases, showing a direct association with the severity of renal injury [40]. Previous observations of activated monocytes (CC chemokine receptor 2(+) ED-1(+)) in peripheral blood, coupled with the infiltration of ED-1(+) macrophages and the increase nuclear p65 in the kidneys of MI rats, have suggested the involvement of NF-κB-mediated inflammation in the development of type 1 cardiorenal syndrome [5,7,9]. Vagus nerve stimulation has been shown to reduce the expression of cytokines and chemokines, including CCL2, a potent chemokine which attracts monocytes/macrophages, and this is accompanied by a decrease in the number of infiltrating macrophages in cisplatin-induced nephropathy [41]. However, the protective effects of cholinergic agonists may also depend on the activation of α7nAChR present on immune cells, as cholinergic stimulation attenuated renal ischemia-reperfusion injury in vagotomized rats [19,42].

The cholinergic-mediated decrease in inflammatory kidney gene expression was accompanied by an upregulation in IL-10 at 7 days, and an upregulation in IL-13 at 30 days, indicating both an acute and a residual anti-inflammatory and immunomodulatory effect of the PY treatment. Cytokine IL-13 plays a crucial role in the polarization of macrophages/dendritic cells to an M2 phenotype, which is essential for recovery from acute kidney injury [43]. The suppression of pro-inflammatory gene expression, alongside the enhanced expression of anti-inflammatory mediators, provided a molecular basis for the immunomodulatory role of PY, which was reflected in the observed reduction of inflammation and tissue preservation. In PY-treated animals evaluated 30 days post-AMI, the levels of NGal, a marker of subclinical renal damage [23], remained closer to the baseline values. Serum creatinine is less sensitive than urine NGAL for diagnosing subclinical AKI stages. Previous studies demonstrated that serum creatinine levels alter after several weeks of AI in rats, while increases in plasma and urine NGAL were detected days after AMI [8]. Indeed, among STEMI patients undergoing primary PCI, elevated NGAL levels are associated with adverse renal and cardiovascular outcomes, independent of traditional inflammatory markers [44]. As stated, vagus nerve stimulation, even after injury, ameliorates cisplatin-induced nephropathy, as detected by a decrease in the expression of subclinical kidney damage biomarkers [41]. Our findings indicate that renal integrity may be better preserved under PY treatment, an important consideration given the renal complications often accompanying AMI. The persistent influence of PY on gene expression profiles, particularly in modulating the balance between pro- and anti-inflammatory cytokines, offers a promising avenue for therapeutic development, warranting further investigation into its mechanisms of action and potential clinical applications.

This article describes the disease phenotype and elucidates the beneficial response to cholinergic stimulation within a cardio-renal milieu post-AMI. However, our investigation did not extend to the mechanisms potentially implicated in this process, including the TGF-β/NF-kB pathway, nor did it quantify cytokine protein levels in renal tissue. Consequently, these aspects represent the limitations of our current study. Future research will be imperative to explore the diverse mechanisms associated with the observations presented herein.

## 4. Materials and Methods

### 4.1. Chemicals and Reagents

Pyridostigmine bromide (PY) was procured from Sigma-Aldrich^®^ (Saint Louis, MO, USA) and was prepared fresh for administration by dissolving in sterile, pyrogen-free phosphate-buffered saline (PBS) sourced from Gibco^®^, Life Technologies (Grand Island, NY, USA). The anesthetic agents, ketamine and xylazine, were obtained from Henry Schein Animal Health (Dublin, OH, USA) and Akorn Animal Health (Lake Forest, IL, USA), respectively. All other chemicals and reagents used were of an analytical grade and were obtained from reputable suppliers.

### 4.2. Animals

Adult male spontaneously hypertensive rats (SHRs) (2–3 months, 200–250 g) were obtained from the vivarium at University Nove de Julho, São Paulo, Brazil. All animals were allowed to acclimate for one week before experimentation. Rats were housed in standard polypropylene cages with a maximum of four animals per cage. The vivarium was carefully monitored, maintaining a controlled environment with 50–60% relative humidity, ambient temperature of 22 to 24 °C, and a 12 h light-dark cycle. Rats had ad libitum access to water and a standard rodent chow diet (Nuvilab, Nuvital brand, containing 12% protein).

The study protocol was reviewed and approved by the Institutional Animal Care and Use Committee (IACUC) of the University Nove de Julho (UNINOVE, reference number 5819150819), ensuring the adherence to ethical standards consistent with The Guide for the Care and Use of Laboratory Animals by the National Academy of Sciences, as published by the National Institutes of Health (NIH). The experimental design and reporting followed the ARRIVE guidelines to enhance the robustness and reproducibility of the results.

### 4.3. Experimental Design

Rats were randomized into three experimental groups: sham-operated controls (Sham), myocardial infarcted rats treated with vehicle (AMI + Veh), and myocardial infarcted rats treated with pyridostigmine (AMI + PY). The sample size for the experimental group was set at 20–24 animals to ensure statistical validity. The AMI + PY group received an oral administration of 40 mg/kg pyridostigmine bromide, delivered via oral gavage starting one hour after AMI induction and continuing once daily for seven days. This therapeutic regimen was based on empirical evidence from a prior investigation which indicated that this dosing strategy attenuates plasma acetylcholinesterase activity by approximately 40% [20]. The AMI + Veh cohort was administered as an equal volume of vehicle to maintain consistency in the experimental procedure.

To elucidate the temporal dynamics of PY administration following AMI on renal pathology, two distinct cohorts, each containing 10–12 animals, were established. The first group was euthanized seven days post-AMI surgery to facilitate acute renal effect analysis. The second group was similarly treated for 7 days, but was allowed a prolonged post-operative period until day 30 post-AMI surgery to assess the sustained effects of cholinergic modulation on renal health and inflammatory processes. Rats were euthanized according to the requirements of the subsequent analytical techniques.

For immunohistochemical studies, 5–6 rats from each cohort were sedated with ketamine (80 mg/kg) and xylazine (12 mg/kg) intraperitoneally (I.P.) and subsequently perfused intravenously (I.V.) with a 0.9% saline solution containing 14 mmol/L KCl at a pressure of 13 cm H2O to induce cardiac arrest in diastole. This was followed by perfusion with 4% buffered formalin to preserve tissue architecture. A subset of 5–6 rats per group were decapitated on the seventh day after thoracotomy for the collection of fresh renal tissue intended for gene expression analysis.

### 4.4. Myocardial Infarction

Prior to the experiment, AMI rats were anesthetized with ketamine (80 mg/kg, i.p.) and xylazine (12 mg/kg, i.p.). AMI was induced after a left thoracotomy through the third intercostal space to reveal the cardiac structure, then the left coronary artery was ligated using a 6.0 mm nylon suture approximately 1 mm distal to the left atrial appendage. The Sham cohort underwent a similar thoracotomy without arterial occlusion under anesthesia, as described for AMI rats. Postoperative management included thoracic closure and vigilant monitoring throughout the recovery phase. All animals received appropriate analgesia post-surgery and were observed for indicators of discomfort, infection, or other complications. Monitoring occurred at regular intervals to ensure adherence to humane research practices.

### 4.5. Arterial Catheterization and Cardiovascular Assessments

One day prior to euthanasia, on days 6 or 29 post-AMI or sham surgery (depending on the cohort), rats underwent a minor surgical procedure under anesthesia, using a combination of 80 mg/kg ketamine and 12 mg/kg xylazine (I.P.) for the implantation of an intra-arterial catheter into the femoral artery for direct recording of arterial blood pressure curves.

The day after catheterization, on day 7 and 30 post-AMI or sham surgery (depending on the cohort), hemodynamic measurements were recorded for 30 min in conscious, awake animals. The arterial cannula was interfaced with a strain gauge transducer (Blood Pressure XDCR; Kent Scientific, Torrington, CT, USA) and the signals were digitized using a data acquisition system (WinDaq, 2 kHz; DATAQ, Springfield, OH, USA).

Cardiovascular variability was assessed through time and frequency domain analyses. Time series for pulse interval (PI) and systolic arterial pressure (SAP) were interpolated and decimated to provide uniform temporal spacing after detrending. Power spectral density analyses were conducted using Fast Fourier transformation, with spectral power for low (LF, 0.20–0.75 Hz) and high frequency (HF, 0.75–4.0 Hz) bands determined by integrating the power spectrum density within each frequency range. The square root of the mean squared differences between adjacent normal PI intervals (RMSSD), and the variance of PI (VAR-PI) and SAP (VAR-SAP), were computed as time domain metrics. The baroreflex sensitivity was assessed using the alpha-index in the low-frequency band, based on the magnitude of squared coherence between PI and SAP signals.

### 4.6. Echocardiographic Evaluation

Echocardiographic exams were performed by a sonographer who was blinded to cohort assignments, conforming to the guidelines of the American Society of Echocardiography. Rats were anesthetized (80 mg/kg ketamine and 12 mg/kg xylazine, I.P.), and images were obtained with a 10–14-MHz linear transducer in a G.E. Vivid 7 Ultra-Definition Clarity Control (G.E. Healthcare, Chicago, IL, USA). This procedure was performed 6 days or 29 days after AMI or sham surgeries in order to analyze AMI area (hipo or acinetic ventricular areas) and LV ejection fraction (LVEF%), and to calculate the following parameters: left atrial diameter (LAD); left ventricular mass (LV M); left ventricular end-diameter during systole and diastole (LVSD, LVDD); E wave A wave ratio (E/A); isovolumetric relaxation time (IVRT); fractional area change (FAC), as described in detail previously in the work presented in [20,21].

### 4.7. Renal Function Marker Analysis

Plasma creatinine concentrations were determined following the methodologies standardized by the Biochemistry Laboratory of the Central Laboratory of InCor, FMUSP. This evaluation was reserved for the cohort observed for 30 days post-surgery to discern long-term renal function alterations.

### 4.8. Histological Examination and Quantitative Collagen Analysis

Renal tissue processing for histological evaluation was conducted on 5–7 animals from each group. Post-euthanasia, tissues were fixed via perfusion with 4% formalin. The tissues were then dehydrated, cleared, and embedded in Paraplast^®^. Longitudinal sections of 5 µm thickness were sliced from the median area of the right kidney. These sections were then deparaffinized, rehydrated, and stained with Picrosirius red for collagen fiber quantification. Images were acquired using a Leica microscope (Leica QWin V3 plus Microsystems Ltd., Cambridge, UK) and an Olympus camera at 200× magnification (Olympus BX-5, Japan Co., Tokyo, Japan). Fifteen consecutive, non-overlapping fields were examined by an observer blinded to the experimental conditions to calculate the percentage of collagen.

### 4.9. Immunohistochemistry for Immune Cells and NGAL

On days 7 and 30 post-AMI surgery or sham surgery, 5–7 animals from each group were anesthetized and perfused as previously described for tissue fixation. All kidney sections were then prepared for CD68. Neutrophil gelatinase-associated lipocalin (NGAL) immunostaining was performed only in animals euthanized on day 30. The process included antigen retrieval (EDTA, pH 8.0; Sigma-Aldrich), the blocking of nonspecific signals with antibody diluents (Antibody Diluent, cat. no. S0809; Dako, Glostrup, Denmark), incubation with primary mouse monoclonal anti-rat CD68 antibody (ED-1 clone, cat. no. 31630, Abcam, Waltham, MA, USA), or anti-Lipocalin-2/NGAL (also known as neutrophil gelatinase-associated lipocalin) (rabbit monoclonal cat. no. ab21092, Abcam), followed by appropriate secondary antibodies, and colorimetric detection. Negative control (in which the primary antibodies were replaced with 1% PBS/BSA and nonimmune mouse serum (X501-1, Dako) were performed to ensure specificity.

An investigator blinded to the cohort samples analyzed the images and manually counted the numbers of CD68+ and NGAL+ cells. Briefly, fifteen consecutive fields (magnification: 400×) in the renal cortex area were photographed with a fluorescence Microscope (Olympus AX70) with a digital camera (Olympus Japan Co., Tokyo, Japan) and the numbers of positive cells were counted manually using Image J software version 1.48v 17 (free software, NIH, Bethesda, MD, USA, EUA) and the “cell counter” plug-in.

### 4.10. Real-Time Quantitative PCR

Total RNA from the left kidney was extracted using TRIzol Universal reagent (Invitrogen, CA, USA), with RNA quantity and quality assessed via NanoDrop spectrometry (ND-2000 spectrometer, Wilmington, DE, USA). cDNA synthesis was performed using the SuperScript III system according to the recommendations of the manufacturer. Quantitative real-time polymerase chain reaction (RT-qPCR) was conducted with PowerUp™ SYBR^®^ Green Master Mix (Applied Biosystems, Austin, TX, USA) on a 7500™ Real-Time PCR System (Applied Biosystems, Waltham, MA, USA). Specific primers were designed using the NCBI (National Center for Biotechnology Information) Primer-Blast for the target cytokines (Table 4) and normalized against β-actin expression. Gene expression data were analyzed using the 2^−∆∆Ct^ method to calculate the relative fold expression.

### 4.11. Statistical Analysis

All results were expressed as the means ± the standard deviation (SD) or the standard error of the mean (SEM), as indicated in the figure legends and tables. Parametric data were analyzed with two-way ANOVA followed by Tukey’s multiple comparisons test, using GraphPad Prism software version 9.0.1 (GraphPad Software, San Diego, CA, USA). A *p*-value of <0.05 denoted statistical significance.

## 5. Conclusions

In this study, we report the cardioprotective and renal anti-inflammatory effects of PY treatment in SHRs subjected to AMI. Our findings provide a compelling rationale for exploring PY and other cholinergic agents as potential therapeutic agents in managing renal inflammation and injury post-AMI. Optimizing the timing and dosage of PY and other cholinergic modulators and further insights into the mechanisms underlying the beneficial effects of cholinergic stimulation in the context of renal injury post-AMI will inform novel efficacious treatments, which are critically needed.

## Figures and Tables

**Table 1:** Hemodynamic parameters of the SHAM, AMI + Veh and AMI + PY groups evaluated at 7 and 30 days of the protocol.

	SHAM 7 Days (*n* = 10)	AMI + Veh 7 Days (*n* = 10)	AMI + PY 7 Days (*n* = 10)	SHAM 30 Days (*n* = 10)	AMI + Veh 30 Days (*n* = 10)	AMI + PY 30 Days (*n* = 10)
SBP mmHg Mean (SD)	203.4 (6.9)	74.8 *^a^ (17.5)	163.3 *^b^ (9.2)	212.5 (8.2)	207.2 (12.5)	189.3 *^c^#^d^ (13.4)
DBP mmHg Mean (SD)	143.7 (7.2)	129.0 *^e^ (8.3)	119.6 *^f^#^g^ (7.9)	148.5 (6.0)	144.4 (6.3)	134.2 (8.2)
MBP mmHg Mean SD)	171.1 (5.6)	148.8 *^h^ (13.9)	142.4 *^i^ (8.7)	176.3 (7.0)	174.8 (9.5)	161.8 *^j^#^k^ (10.0)
HR bpm Mean (SD)	373.5 (26.1)	393.0 (22.1)	375.7 (26.7)	374.4 (26.3)	384.3 (22.1)	369.0 (11.9)

SBP = systolic blood pressure; DBP = diastolic blood pressure; MAP = mean arterial pressure; HR = heart rate; SD = standard deviation of the mean. * = group vs. SHAM # = group vs. AMI + Veh. The *p*-value for: a = <0.001, b = <0.001, c = <0.001, d = 0.054, e = 0.008, f = <0.001, g = 0.032, h = <0.001, i = <0.001, j = 0.003, k = 0.008.

**Table 2:** Components of heart rate and blood pressure variability, and baroreflex sensitivity of the SHAM, AMI + Veh and AMI + PY groups evaluated at 7 and 30 days of the protocol.

	SHAM 7 d (*n* = 10)	AMI + Veh 7 d (*n* = 8)	AMI + PY 7 d (*n* = 10)	SHAM 30 d (*n* = 10)	AMI + Veh 30 d (*n* = 10)	AMI + PY 30 d (*n* = 10)
HRV						
RMSSD (ms) SD	6.2 (2.0)	5.8 (2.2)	9.1 *# (2.1)	8.1 (2.9)	9.8 (2.7)	10.1 (2.0)
VARPI (ms^2^) SD	28.5 (13.0)	15.7 (6.0)	60.1 *# (27.9)	51.5 (18.7)	69.6 (30.6)	91.8 * (49.0)
LF ab (ms^2^) SD	2.5 (1.9)	2.5 (1.0)	7.2 *# (4.6)	2.2 (1.4)	2.6 (1.4)	3.9 (2.0)
HF ab (ms^2^) SD	9.6 (3.5)	5.9 (2.9)	22.7 *# (9.0)	25.6 (17.5)	26.3 (12.8)	30.1 (11.0)
LF (nu) SD	19.3 (7.3)	31.7 * (9.8)	22.9 (6.9)	10.1 (3.1)	11.1 (4.0)	12.0 (2.9)
HF (nu) SD	80.6 (7.3)	68.3 * (9.8)	77.1 (6.9)	89.5 (2.7)	88.5 (4.0)	87.5 (3.8)
LF/HF SD	0.2 (0.1)	0.5 * (0.2)	0.3 (0.1)	0.11 (0.03)	0.12 (0.04)	0.14 (0.05)
SAPV						
VAR-SBP (mmHg^2^) SD	57.8 (18.5)	28.6 * (12.1)	30.4 * (14.2)	41.2 (8.1)	43.0 (18.4)	47.6 (19.3)
LF-SBP (mmHg^2^) SD	14.7 (8.2)	11.1 (8.0)	8.7 (4.5)	11.1 (3.0)	12.1 (4.5)	7.8 # (2.8)
ALFA INDEX ms^2^/mmHg^2^ SD	0.49 (0.34)	0.55 (0.19)	0.98 *# (0.48)	0.44 (0.15)	0.57 (0.17)	0.70 * (0.26)

HRV = heart rate variability; RMSSD = root mean square of successive differences between normal heart beats; VARPI = absolute power of the heart rate variability; LF ab = absolute power of the low-frequency band (0.04–0.15 Hz); HF ab = absolute power of the high-frequency band (0.15–0.4 Hz); LF (nu) = Relative power of the low-frequency in normal units; HF (un) = relative power of the high-frequency band in normal units; LF/HF = ratio of LF-to-HF power; SAPV = systolic arterial pressure variability; VAR-SBP = absolute power of blood pressure variability; LF-SBP (mmHg^2^) = absolute power of the low-frequency band of systolic blood pressure variability; ALFA INDEX = power ratio of RR interval variability and of systolic arterial pressure series variability. * = *p* < 0.05 group vs. SHAM # = *p* < 0.05 group vs. AMI + Veh.

**Table 3:** Structural and functional echocardiographic parameters of the SHAM, AMI + Veh and AMI + PY groups evaluated at 7 and 30 days of the protocol.

	SHAM 7 Days (*n* = 10)	AMI + Veh 7 Days (*n* = 10)	AMI + PY 7 Days (*n* = 10)	SHAM 30 Days (*n* = 10)	AMI + Veh 30 Days (*n* = 10)	AMI + PY 30 Days (*n* = 10)
PARAMETER						
LAD (mm) SD	4.25 (0.95)	4.58 (1.19)	4.08 (0.70)	5.11 (0.67)	5.52 (0.66)	5.18 (0.50)
LVEF (%) SD	51.48 (8.54)	33.62 * (13.17)	37.19 * (10.59)	53.69 (6.76)	39.54 * (6.49)	29.21 * (6.50)
LVFAC (%) SD	43.9 (5.9)	28.02 * (7.11)	32.04 * (9.17)	47.91 (6.72)	28.11 * (6.27)	25.26 * (3.27)
LVSD (mm) SD	5.64 (0.76)	7.12 * (0.60)	6.06 # (1.21)	6.30 (0.80)	7.54 * (0.64)	8.18 * (0.57)
LVDD (mm) SD	7.77 (0.61)	8.53 * (0.92)	7.44 # (1.02)	8.76 (0.38)	9.70 * (0.63)	9.44 * (0.26)
LV M (mg) SD	460.2 (59.52)	522.7 (123.1)	447.6 (142.7)	591.2 (112.7)	550.1 (82.42)	531.9 (68.57)
IVRT (ms) SD	18.25 (3.11)	17.59 (3.71)	20.53 (4.06)	17.24 (3.95)	19.58 (2.12)	24.62 * (6.64)
E/A Ratio SD	1.42 (0.12)	2.12 * (0.62)	1.47 # (0.18)	1.91 (0.67)	2.15 (0.76)	1.72 (0.30)
E′/A′ Ratio SD	0.69 (0.16)	0.98 (0.37)	1.28 * (0.53)	1.04 (0.39)	0.87 (0.26)	1.05 (0.28)
E/E′ Ratio SD	24.89 (5.84)	30.09 (9.59)	18.15 # (3.44)	23.87 (8.72)	31.84 (8.69)	31.86 (8.19)

LAD = left atrial diameter; LVEF = left ventricular ejection fraction; LVFAC = left ventricular fractional area change; LVSD = left ventricle systolic diameter; LVDD = left ventricle diastolic diameter; LV M = left ventricular mass; IVRT = isovolumetric relaxation time (IVRT); E = early filling component of mitral inflow pattern; A = late filling component of mitral inflow pattern; E′ = tissue Doppler imaging of E wave; A′ = tissue Doppler imaging of A wave. * = *p* < 0.05 group vs. SHAM # = *p* < 0.05 group vs. AMI.

**Table 4:** Primer sequences used for gene expression analysis.

Cytokine	Sense	Antisense
TGF-β1	CTGCTGACCCCCACTGATAC	AGCCCTGTATTCCGTCTCCT
MCP-1	GCTGCTACTCATTCACTGGCAA	GCTGCTGGTGATTCTCTTGTA
IL-1β	AAATGCCTCGTGCTGTCTGA	GCTGTTTTAGGGACACCGGA
TNF-α	CTCAAGCCCTGGTATGAGCC	CTCCAAAGTAGACCTGCCCG
IL-6	AGCGATGATGCACTGTCAGA	GGAACTCCAGAAGACCAGAGC
IL-13	CCTGGAATCCCTGACCAACA	ATCCGAGGCCTTTTGGTTACA
IL-10	TTGAACCACCCGGCATCTAC	CCAAGGAGTTGCTCCCGTTA
ß-actin	AGGAGTACGATGAGTCCGGCCC	GCAGCTCAGTAACAGTCCGCCT

## Data Availability

Original data may be shared upon request.
